# Growing up with chronic traffic noise exposure leads to transient but not long-term noise tolerance in a songbird

**DOI:** 10.1098/rsbl.2024.0575

**Published:** 2025-01-08

**Authors:** Quanxiao Liu, Hans Slabbekoorn, Katharina Riebel

**Affiliations:** ^1^ Department of Zoology, Stockholm University, Stockholm, Sweden; ^2^ Institute of Biology Leiden, Leiden University, Leiden, The Netherlands

**Keywords:** anthropogenic noise, noise avoidance, traffic noise, avian development, noise pollution

## Abstract

Noise pollution is on the rise worldwide. An unresolved issue regarding the mitigation of noise pollution is whether and at which timescales animals may adapt to noise pollution. Here, we tested whether continuous highway noise exposure perinatally and during juvenile development increased noise tolerance in a songbird, the zebra finch (*Taeniopygia castanotis*). Breeding pairs were exposed to highway noise recordings from pre-egg-laying until their offspring reached subadulthood. Subsequently, offspring were tested for noise tolerance both as subadults and adults in a spatial preference test, where birds could choose to enter aviaries with different levels of highway noise. Unlike control birds that preferentially chose the quiet aviaries, noise-reared birds exhibited no spatial preferences for quiet in the first test. However, when the experimental birds were retested after two months without noise exposure, they now avoided the previously tolerated noise levels and preferred the quieter aviary. The increased noise tolerance observed directly after the release from the noise treatment was thus only transient. Growing up with chronic highway noise exposure did thus not increase subjects’ noise tolerance, meaning that at least in this songbird species, adaptation to noise pollution is unlikely to arise on a developmental time scale.

## 1. Introduction

Traffic noise is now a widely recognized environmental pollutant. Habitats with high levels of anthropogenic noise often have reduced or altered species composition [1–5]. Active noise avoidance has been hypothesized to be one of the drivers of biodiversity loss and population declines along roads [6–9]. For several bird species, playback experiments have now demonstrated that playback of traffic noise *per se* can deter birds from previously visited locations or even breeding sites [10–14]. It is important to note that these experiments either tested laboratory birds without prior experiences with the type of tested noise [13,14] or wild birds of unknown background [10–12]. This leaves the question of whether growing up with traffic noise increases noise tolerance unresolved [1,15–17].

Chronic noise exposure could induce stronger noise tolerance in different ways. In extremis, high noise levels could damage a bird’s hearing system, resulting in temporary or permanent threshold shifts and thus changing noise tolerance levels due to reduced ability to perceive acoustic signals [15,18]. Noise could also lead to experience-dependent behavioural changes because of habituation or sensory adaptation, manifesting as reversible, decremental behavioural responses to noise [19]. This has not been tested experimentally but seems likely given the many behavioural and physiological effects of increased noise exposure during development [20–25], which in turn could affect how birds react to noise [16,26]. As the potential for increased tolerance from early experience is often discussed [16,27,28], but not empirically tested, we here investigated whether continuous noise exposure during development results in more noise-tolerant birds, and if so, at what time scale such tolerance would persist.

Zebra finches are a suitable model for such an experimental study. Their development is well studied [29] and their hearing curves and thresholds for auditory damage are well characterized [30–32]. Moreover, we recently developed and validated a two-way spatial preference paradigm for zebra finches that allows birds to move freely between different acoustical environments [14]. With this paradigm, birds can actively indicate their preferences and adult zebra finches from our colony showed differentiated noise avoidance behaviour towards different types of highway noise. With avoided versus tolerated noise levels established in these previous tests [14], the tested adult birds were recruited as breeding pairs for the current study. If continuous perinatal and juvenile noise exposure affected noise tolerance levels, noise-reared offspring should behave differently from same-age control birds reared without noise in the spatial noise avoidance tests. If growing up in traffic noise affects birds’ phenotypes permanently, they should show higher noise tolerance than their parents, both as subadults and adults later in life.

## 2. Material and methods

### (a) Subjects

Subjects were wild-type outbred zebra finches from the Leiden University breeding colony. Thirty breeding pairs reared 109 chicks (57 females/52 males) in two breeding rounds with two types of continuous noise exposure (see below). An additional 28 subadults (19 females/nine males, from nine breeding pairs) were raised without experimental noise exposure as a control group. All offspring were housed with their parents in breeding cages (1 × 0.5 × 0.4 m). The breeding pairs and their offspring in the two noise exposure treatments were housed in one of two identical rooms (3.65 × 3.05 × 3 m) until the offspring reached 65 ± 4 days old. Both rooms had a 14 : 10 h light : dark light regime with a temperature of 20−22°C and humidity of 35–50%. The birds from the control group were raised in the colony room in the same breeding cages with the same climate and lighting settings. Birds had *ad libitum* access to water, mixed seeds, grit and cuttlefish bone, complemented twice a week with egg food, fruit and vegetables, and once a week with germinated seeds. Pairs in experimental groups were exposed to both moderate-intensity (previously not avoided) and high-intensity (avoided) continuous highway noise in two breeding rounds in a fully balanced design with crossover.

### (b) Highway noise stimuli

Birds received continuous playback of 24 h recordings from Dutch highways [14]. The noise levels during breeding were chosen based on the outcomes of the previous tests with the parents themselves, who had avoided the high-intensity noise (55–75 dB(A) re: 20 μPa, recorded at 5–15 m distance from a busy highway) but tolerated the moderate-intensity noise (45–55 dB(A), recorded at 200–400 m distance). In comparison, noise levels in the colony room were from 35 to 41 (ambient noise) and to 61 dB(A) (loud bird calls).

### (c) Noise avoidance test

We used the same set-up as [14] had used to test the (then noise naive) parents, to now test their offspring’s noise avoidance. The set-up (for details see electronic supplementary material, figure S2) consisted of two aviaries (2 × 2 × 2 m) that were interconnected by a wire mesh flight corridor (0.5 × 0.5 × 1 m). To fly from one aviary to the other, birds had to pass the corridor with a smaller (30 × 30 cm) opening in the middle, which held an antenna (ANTSER300, Dorset, Aalten, The Netherlands) that registered each passing bird via the ID tag on their leg rings. In each aviary, one loudspeaker (CB4500, Blaupunkt, Hildesheim, Germany) was placed in the corner furthest away from the opening to the flight tunnel.

As subadults (65 ± 4 dph, days post hatch), birds were tested in small groups of on average four birds (30 groups with four birds, but three groups with three birds, and one group of two birds) that were either all-female (*N*_f_ = 6), all-male (*N*_m_ = 1) or mixed (*N*_mix_ = 29) to avoid stress from isolation in this social species. For each test, a group was first moved from their home cages into the set-up between 15.00 and 16.00 h and then left to acclimate for the next 18 h to explore the set-up until testing started between 09.00 and 10.00 h the next morning. During acclimation, sound exposure was the same as in subjects’ respective rearing rooms. After the first test, all experimental birds were released from their respective noise treatments and moved to standard housing in one of the unisex aviaries of the colony (1 × 2 × 2 m) and retested as young adults two months later (115 ± 17 dph). Testing now occurred in single-sex groups only (*N*_f_ = 15, *N*_m_ = 9) of three or four birds (*N*_4_ = 19, *N*_3_ = 5) to prevent courtship behaviour in the test. Birds now had no playback during acclimation. For logistic reasons (there was only one set-up to test all birds from the two treatments and two breeding rounds) and because our main interest was to test whether the increased noise tolerance of the noise-exposed juveniles would carry over into adulthood, the noise-naive subadult control birds were not retested in adulthood as they had shown the same noise avoidance as the naive adults of the parental generation previously tested with the same stimuli [14].

Each test (followed [14], for additional information, see electronic supplementary material) started with a 15 min baseline observation phase (‘pre-playback’), followed by 2 × 30 min playback (high- or moderate-intensity) exposing the birds first to noise playback in one aviary but not in the other for 30 min, and then another 30 min with the noise conditions reversed between sides. After a 15 min silent break, another 2 × 30 min playback session started, but birds that had first been tested with the high-intensity noise now received the moderate-intensity noise and *vice versa*. After another 15 min silent break, the high- and moderate-intensity noise stimuli were now played back simultaneously for 30 min (one noise level in each aviary) before sounds were switched between sides and played back for another 30 min. All noise playbacks began and ended with a 2 min fade-in and fade-out to avoid startling the birds by sudden noise onsets.

During all trials, an antenna registered the electronic leg ring tags passing through the tunnel, thus counting the switches between aviaries to facilitate video analyses. The tunnel was continuously filmed (webcam HD Pro C920, Logitech, Lausanne, Switzerland) to record all bird transitions between the aviaries. Videos were checked by QL (sound turned off and analysing videos with non-informative IDs to ensure that the analysis was conducted double-blinded), using BORIS v. 6.1.6 [33]. The number of transitions between the two aviaries and the total time spent by bird groups in either aviary during each phase of the experiment were scored cumulatively. Time spent could range from 0 min (not a single bird visited) to 240 min (4 × 60 min, if all four birds stayed the whole time in the same aviary).

### (d) Analyses

All statistical analyses were conducted in R 3.5.2 with tested groups as the unit of analysis (as birds within the small test group cannot be assumed to move independently of each other). As the proportion of time spent per aviary was bounded, this parameter was arcsine square-root transformed to ensure data distributions met the criteria of a normal distribution. We then first tested whether there were any side preferences for one of the two aviaries during the pre-playback phase using a one-sample *t*‐test against the expectation of (arcsine square-root transformed) 0.5. Then, we used a one-way ANOVA with rearing noise condition as the only factor and the proportion of time spent in the left aviary as the response variable to test if there were differences among groups. We then tested whether rearing noise levels affected time spent in the quiet aviary during the ‘noise versus quiet’ phase with a mixed linear model with the proportion of time spent in the quiet aviary as the response variable, rearing condition, noise playback type, and their interaction as fixed factors, and ‘group ID’ and ‘stimuli set’ as random intercepts (table 1, Model A, ‘lmer’ function from package ‘lme4’). We explored the effects of playback order interacting with playback types; however, adding this interaction or order alone did not improve the model. Using least-squares means (EMMs), we compared the three rearing conditions *post hoc* (electronic supplementary material, table S1, Model A, ‘emmeans’ function from package ‘emmeans’). To test whether the moderate-intensity aviary was preferred during the simultaneous ‘high versus moderate’ phase, the effects of rearing conditions were tested using a linear model (table 1, Model B, ‘lm’ function from package ‘stats’). EMMs were computed for *post hoc* analyses of the differences among all three rearing conditions (electronic supplementary material, table S1, Model B). We applied the same statistical methods to the data from the second test round, but then with two rearing conditions (high/moderate).

**Table 1.** Linear mixed-model analysis of the time spent in the quieter aviary by subadult zebra finch groups from control, moderate- and high-intensity background noise rearing conditions.

noise versus quiet subadult^a^	χ^2^	d.f.	***p*-value**
rearing condition	12.53	*2*	**0.002**
playback type	5.86	1	**0.016**
rearing condition × playback type	2.46	2	0.293

moderate versus high subadult^b^	sum sq	d.f.	***p*-value**
rearing condition	0.10	2	0.074

Statistically significant results (*p* < 0.05) are highlighted in bold.

^a^ *R*^2^_conditional_ = 0.49.

^b^ *R*^2^_marginal_ = 0.24.

## 3. Results

**Figure 1.**
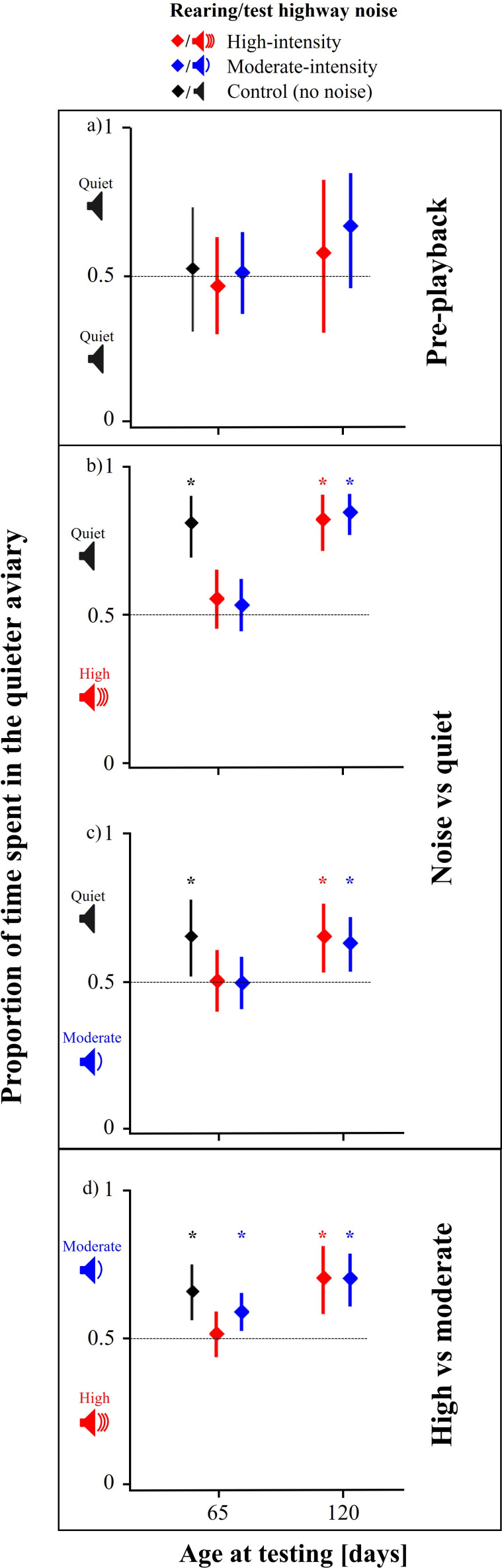
Noise avoidance behaviour by zebra finches in relation to levels of noise exposure experienced during development. (*a–d*) Mean proportion of time spent in the quieter of the two aviaries during different playback conditions and at different ages (diamonds = mean of all groups tested, bars = 95% CI). Sample sizes at 65 days (120 days): reared in moderate-intensity noise = 17 (14) groups, high-intensity noise = 12(10) groups, control = seven groups. Red: high-intensity noise, blue: moderate-intensity noise, black: control. *Y*-axis: loudspeaker symbols and colours indicate noise playback conditions. (*a*) Quiet versus quiet (pre-playback), (*b*) high versus quiet, (*c*) moderate versus quiet, (*d*) high versus moderate. The aviary and noise types were balanced within and across treatment groups. * Significantly different from 0.5 chance level.

In the first test, subadult birds from all groups (*N*_control_ = 7, *N*_moderate_ = 17, *N*_high_ = 12 groups) used both aviaries equally often during the pre-playback phase (control group *t*_6_ = 0.29, *p* = 0.78; moderate-intensity *t*_16_ = 0.11, *p* = 0.91; high-intensity *t*_11_ = −0.47, *p* = 0.65), and there were no differences between treatments (F_2, 33_ = 0.13, *p* = 0.88, figure 1*a*). This changed during the noise playbacks: the control birds actively moved away from noise playbacks towards the quiet aviaries (table 1, figure 1*b*,*c*) and this avoidance was also present during the high- versus moderate-intensity traffic noise playbacks (electronic supplementary material table 1). In contrast, the noise-reared birds continued using and staying in both aviaries even if in one of the aviaries they were exposed to high-intensity traffic noise (table 1, figure 1*b*,*c*). During the high- versus moderate-intensity traffic noise playbacks, control subadults and subadults raised in moderate-intensity traffic noise avoided high-intensity traffic noise. In contrast, subadults raised in high-intensity traffic noise did not avoid high-intensity noise and spent similar amounts of time in both aviaries (electronic supplementary material, table S1, figure 1*c*). All groups actively switched between aviaries under all test conditions, and rearing conditions did not affect the total number of transitions between two aviaries (electronic supplementary material, figure S1).

Two months after the first test, when the birds from the two noise treatments (*N*_moderate_ = 14, *N*_high_ = 10 groups) had matured into young adults, they were retested with the same playback procedure and stimuli as before. Again, birds used both aviaries equally often during pre-playback (moderate-intensity rearing group *t*_13_ = 0.41, *p* = 0.68; high-intensity *t*_9_ = −0.50, *p* = 0.62) and there were no differences between treatments (F_1, 22_ = 0.31, *p* = 0.58, figure 1*a*). In contrast to the first test, the experimental birds now avoided noisy aviaries in favour of the quieter options and more strongly so for the high-intensity traffic noise playback (table 2, figure 1*b*,*c*). Likewise, birds from both treatments also avoided the high-intensity traffic noise during the simultaneous playbacks (figure 1*d*). Interestingly, the noise-exposed birds now avoided not only the high-intensity noise like their parents but also the moderate-intensity noise that was previously tolerated by their parents (see electronic supplementary material, figure S2).

**Table 2.** Linear mixed-model analysis of time spent in the quieter aviary of the tested adult zebra finch groups.

noise versus quiet adult^a^	χ2	d.f.	***p*-value**
rearing condition	0.01	1	0.94
playback type	26.8	1	**<0.001**
rearing condition × playback type	0.42	1	0.52

moderate versus high adult^b^	sum sq	d.f.	***p*-value**
rearing condition	<0.001	1	0.98

Statistically significant results (*p* < 0.05) are highlighted in bold.

^a^ *R*^2^_conditional_ = 0.54.

^b^ *R*^2^_marginal_ = 0.27.

## 4. Discussion

Our experiment tested whether birds that experienced chronic highway noise throughout perinatal and juvenile phases would show increased noise tolerance later in life, using the same stimuli that their parents had either tolerated (moderate-intensity) or avoided (high-intensity) in spatial preference tests in adulthood [14]. When tested immediately upon release from the noise treatment, noise-reared subadults indeed showed a higher noise tolerance than their parents and same-age control birds. In contrast, the same noise-reared birds avoided both the previously tolerated high- and moderate-intensity highway noise when retested as young adults two months later. It is particularly interesting that although these birds had experienced the same type of stimuli from within the egg onwards and had not avoided them as subadults, they now avoided these sounds and that their avoidance behaviour not only aligned with that of naive subadult (controls) and adult birds (their parents) but, if anything, they showed less tolerance as they also avoided the moderate noise levels previously tolerated by their parents. The initially increased noise tolerance was thus only transient and not a lasting manifestation of a more noise-tolerant phenotype and seemed even to have turned into a higher noise sensitivity than that observed in their parents.

The absence of a permanent noise tolerance despite continuous noise exposure during development is congruent with the phenomenon of temporary threshold shift (TTS) in the auditory domain [19,34]. Such temporary shifts in sensitivity can arise through different mechanisms, including sensory or perceptual short-term adaptations or in extreme cases even the loss of hair cells. However, TTS arising from hair cell damage is an unlikely explanation. In our study, the combination of the amplitude and exposure duration was much lower than the reported thresholds for hair cell damage. Peak levels in our study were 77.4 dB(A) during the high-intensity treatment, which was well below 93 dB(A), the estimated level for continuous noise to induce TTS in zebra finches [32,34]. Therefore, the transient noise tolerance observed in the first test seems more likely to have resulted from sensory or perceptual short-term adaptations, but the exact mechanisms underlying the behavioural changes will have to be addressed by neuro-psychophysiological studies [34,35].

Our aim to test whether rearing in noise would lead to noise-tolerant phenotypes was partly motivated by observations of developmental plasticity in sensory systems of other taxa, where organisms can develop into different ‘sensory phenotypes’ that are permanently adaptive to the specifically exposed stimuli (e.g. different colour sensitivities in guppies raised under different light conditions [36,37]). However, we found no evidence that the continuous noise exposure led to more noise-tolerant phenotypes even though our exposure scheme, from pre-breeding to subadulthood, could not have missed critical windows during perinatal development. Our results are however congruent with multiple observations showing that birds react with behavioural flexibility rather than phenotypic change to noise exposure. Birds temporarily change their song amplitude, timing or spectral structure when singing in noisy environments [38–42]. Notably, white-crowned sparrows (*Zonotrichia leucophrys*) with noise-adapted loud and simple songs immediately reversed to softer and more complex songs upon release from traffic noise during the COVID-19 lockdown [43]. This study thus showed that even after generations of noise exposure, changes in singing behaviour (and thus likely also in receiver behaviour) can remain reversible. Birds inhabiting noisy areas often experience fluctuating noise levels [44–46], and a dynamic noise tolerance, increasing with noise exposure and decreasing again with quieter conditions, seems to be how birds currently cope with an increasingly noisy and acoustically unpredictable world [26,47]. The quick reverting in spatial preferences and singing strategies do however suggest that these strategies are potentially suboptimal coping strategies arising from lack of better options, a view that is supported by the many findings showing that noise exposure can result in aversive physiological changes [20,23,25,48,49], which may not manifest directly on the behavioural level, but that may nonetheless affect how birds with different experiences cope with noise upon re-exposure [50,51], a topic that requires further investigation [52,53].

Our tests complement the existing work on noise avoidance tests in birds by investigating developmental influences on noise avoidance behaviour. Experimental work to date that tested behavioural reactions to noise exposure created, for example, ‘phantom roads’ in the outdoor conditions of natural habitats, but thereby tested sounds that were novel to the subjects [11]. Developmental studies that exposed birds to noise from an early age primarily focused on effects on physiology and vocal development [48,54,55], but not on whether the long-term exposure treatments affected noise tolerance afterwards. The results here complement these studies by confirming that loud traffic noise remains aversive to birds even if they have experienced it throughout development. Furthermore, we resolve contrasting results of earlier experiments on noise avoidance-based spatial preferences. Noise playbacks in two earlier studies showed aversive effects of noise on the spatial use of zebra finches [13,14], but in another study, zebra finches did not show spatial avoidance of high-intensity noise [56]. For the latter, the authors reported and speculated that the high background noise (55–86 dB(A)) in the rearing room might have increased noise tolerance to the stimuli that ranged from 60 to 80 dB(A). The levels in their housing room are comparable in amplitude level to the high-intensity noise treatment in this study. Meaning birds in [56] were actually raised at a noise level that led to a (transiently) increased noise tolerance in our study. If the lack of avoidance in [56] reflected the same elevated but transient noise tolerance, as revealed now in our study, we would expect the same birds to not tolerate the noise after housing in quieter conditions. However, birds were not retested at a later age in their study. Combined, these studies suggest that at least some variation in noise avoidance behaviour is experience driven and that developmental exposure to chronic noise can have nuanced and time-sensitive effects on both noise tolerance and noise avoidance behaviour.

To summarize, perinatal and juvenile exposure to chronic highway noise led to increased noise tolerance directly upon release from the noise treatment, but birds lost this tolerance later in life when they showed the same or even more pronounced noise avoidance than their noise-naive parents had shown in the same type of test. Our study was not designed to identify the exact timepoint when the increased noise tolerance started fading, but the observations allow us to conclude that the effects of the noise exposure during development had different consequences in the short and long term. Future studies are required to identify the mechanism(s) underlying the transient and reversible effect of experience-dependent noise tolerance. We provide evidence that chronic noise exposure during development does not result in permanently more noise-resilient phenotypes via persistently elevated tolerance levels. While the timescale of fading tolerance and the possible existence of fluctuations in noise tolerance later in life require further studies with higher time resolution, our study demonstrates the nuanced and time-sensitive effects of noise tolerance should be factored into potential mitigation measures.

## Data Availability

The original data of this manuscript can be found in the electronic supplementary materials (file ‘Liu et al_chronic traffic noise exposure and noise tolerance.xlsx’). The R scripts for statistical analysis can be found in supplementary material file ‘Liu et al_ chronic traffic noise exposure and noise tolerance_analysis.R’. All electronic supplementary data are available from the Dryad Digital Repository [57]. Electronic supplementary material is available online [58].
